# Spatial age-period-cohort analysis of hepatitis B risk in Xinjiang from 2006 to 2019

**DOI:** 10.3389/fpubh.2023.1171516

**Published:** 2023-05-31

**Authors:** Yijia Wang, Na Xie, Fengjun Li, Zhe Wang, Shuzhen Ding, Xijian Hu, Kai Wang

**Affiliations:** ^1^College of Mathematics and System Science, Xinjiang University, Urumqi, China; ^2^Xinjiang Center for Disease Control and Prevention, Urumqi, China; ^3^Department of Medical Engineering and Technology, Xinjiang Medical University, Urumqi, China; ^4^Zhongshan School of Medicine, Sun Yat-sen University, Guangzhou, China

**Keywords:** risk of hepatitis B, clustering analysis, spatial age-period-cohort model, INLA, spatio-temporal analysis

## Abstract

**Objective:**

The objective of this study was to investigate the spatio-temporal distribution and epidemiological characteristics of hepatitis B in 96 districts and counties of Xinjiang and to give useful information for hepatitis B prevention and treatment.

**Methods:**

Based on the incidence data of hepatitis B in 96 districts and counties of Xinjiang from 2006 to 2019, the global trend analysis method was used to characterize the spatial variability of the disease, and the spatial autocorrelation and spatio-temporal aggregation analysis were used to explore the spatial clustering of hepatitis B and to identify high-risk areas and periods. The Integrated Nested Laplace Approximation (INLA)-based spatial age-period-cohort model was established to further explore the influence of age, period, birth queue effect, and spatial distribution on the incidence risk of hepatitis B, and sum-to-zero constraint was adopted to avoid the issue of model unrecognition.

**Results:**

The risk of hepatitis B in Xinjiang is increasing from west to east and from north to south, with spatial heterogeneity and spatio-temporal scanning statistics yielding five clustering areas. The spatial age-period-cohort model showed two peaks in the average risk of hepatitis B, at [25,30) years old and [50,55) years old, respectively. The mean risk of hepatitis B incidence fluctuated up and down around 1 with time, and the average risk of disease by birth cohort displayed an increasing-decreasing-stabilizing trend. Taking age, period, and cohort effect into consideration, it was found that the areas with a high risk of hepatitis B are Tianshan District, Xinshi District, Shuimogou District, Changji City, Aksu City, Kashi City, Korla City, Qiemo County and Yopurga County in Xinjiang. According to the spatio-temporal effect item, it was found that there are unobserved variables affecting the incidence of hepatitis B in some districts and counties of Xinjiang.

**Conclusion:**

The spatio-temporal characteristics of hepatitis B and the high-risk population needed to be taken into attention. It is suggested that the relevant disease prevention and control centers should strengthen the prevention and control of hepatitis B among young people while paying attention to middle-aged and older adult people, and strengthening the prevention and monitoring of high-risk areas.

## 1. Introduction

Hepatitis B is a Class B infectious disease caused by the hepatitis B virus (HBV). Hepatitis B has the characteristics of strong infectivity, complicated transmission routes, high incidence rate, and high disease burden (1–3). China is a country with a high burden of hepatitis B, and there are about 86 million hepatitis B virus carriers. About 28 million of them are hepatitis B patients who need treatment. The epidemic situation of hepatitis B is very serious, and the northwest of China is a high-incidence area of hepatitis B, and its incidence rate is also rising (4).

The epidemic of hepatitis B has certain spatial and temporal dynamics as a spatial and temporal phenomenon in its occurrence, development, and epidemic (5). It is an important area of study. As early as 1950, Moran’s I index was first proposed by Moran to reflect the spatial clustering degree of attribute variables in the whole research area, but spatial autocorrelation analysis only analyzed the correlation degree of variables in the spatial dimension, without considering the temporal characteristics of clustering. Compared with this, the scanning statistics method proposed by Kulldorff and other scholars showed obvious advantages. Kulldorff et al. cooperated to develop SaTScan software, which made the spatio-temporal scanning method widely used in epidemiological research. Kulldorff (6) proposed spatio-temporal scanning statistics based on the Poisson model and the Bernoulli model. Scanning statistics method can be used to monitor and evaluate the clustering of diseases in time, space, or spatio-temporal. Spatial autocorrelation and spatiotemporal scanning analysis methods are currently in widespread use. Gwentira et al. (7) used spatial autocorrelation analysis and spatio-temporal scanning analysis method to explore the distribution characteristics of malaria in Zimbabwe. Gou et al. (8) used these two methods to analyze the spatial autocorrelation of hepatitis B and the temporal and spatial trends of spatio-temporal clustering in Gansu Province, the study found that the incidence of hepatitis B in Gansu Province was spatially autocorrelation from 2009 to 2014, and the high incidence areas of hepatitis B were mostly concentrated in the Hexi area in 2009–2011, and in 2012–2014. Some scholars also use two methods to study respiratory tuberculosis (9), COVID-19 (10), and other diseases. There is currently no research on the spatio-temporal characteristics of the risk of hepatitis B in the 96 districts and counties of Xinjiang using the two methodologies mentioned above.

Exploring the clustering characteristics of hepatitis B risk can provide some basis for the rational allocation of medical and health resources, but it is impossible to determine the possible determinants of hepatitis B. In a long-term epidemiological study, three internal factors-age, birth cohort, and calendar year (i.e., historical trend)-play a significant impact. Lagazio et al. (11) established a spatial age-period-cohort model and combined it with the INLA method to study the temporal and spatial pattern evolution of disease risk. Age effect has nothing to do with the time period or queue that an individual belongs to; rather, it reflects the risk changes connected to distinct groups of ages, including physiological changes and social experiences related to aging; Period effects, including early risk factors such as socioeconomic environment, demography, and lifestyle, are the outcomes of outside variables influencing all age groups at a certain period in a calendar year; The queue effect is a change in time that all packets experience from their start time (12). Etxeberria et al. (13) used this method to analyze the mortality of pancreatic cancer in 50 provinces of Spain in terms of space, sex, age, period, and birth cohort. To ensure the identifiability of the model, the author applied the sum-to-zero constraint, and this method was also used in this study. Chernyavskiy et al. (14) used the age-period-cohort with spatial changes to explore mortality in the United States. Froelicher et al. (15) used the spatial age-period-cohort method to study the incidence of breast cancer in the Portuguese region. There are many studies employing spatial autocorrelation methods to investigate the geographical distribution of hepatitis B, but none of them take into account the influence of space, age, period, and cohort.

Rue proposed the INLA method for rapid computational Bayesian inference in 2009 (16). Because the components of the latent field and the vector of hyperparameters and latent field are significantly reliant on one another, especially when the dimension n is big, the Markov Chain Monte Carlo (MCMC) approach frequently performs poorly (17). Compared with the MCMC method, the INLA method has more powerful computing capabilities without losing accuracy. Based on this important algorithm, this study combines temporal, cohort, and spatial characteristics to characterize the prevalence of hepatitis B in 96 districts and counties in Xinjiang, which is both a new and meaningful study.

Based on the incidence data of hepatitis B in 96 districts and counties of Xinjiang from 2006 to 2019, this study discusses whether there is spatial clustering in the prevalence of hepatitis B in Xinjiang by using spatial autocorrelation and spatio-temporal clustering analysis methods, identifies the high-risk clustering regions and periods, and establishes a spatial age-period-cohort model based on INLA method to further explore the influence of age, period, birth cohort effect and geographical distribution on the incidence risk of hepatitis B, to provide a scientific basis for the epidemic and control of the disease.

## 2. Materials and methods

### 2.1. Data collection

The data on hepatitis B cases in 96 districts and counties in Xinjiang from 2006 to 2019 was collected from the infectious disease information management system of the Xinjiang CDC. The population data of each district and county are from the Statistical Yearbook of Xinjiang from 2004 to 2019. The data includes the year, age group, incidence risk, incidence number, and location. The number of people in each age structure in each district and county was calculated by the proportion of age structure (0–5, …, 95–100, more than 100) in the sixth national census in 2010. Since the total number of cases over 100 years old is only 34 cases (accounting for 0.006% of the total), the incidence of this age group is deleted for the convenience of data processing and exploration. Finally, the risk of hepatitis B in different age groups in each district and county is calculated, and the formula is as follows.


$$SMR_{ait}=\frac{Y_{ait}}{E_{ait}}$$

where $SMR_{ait}$ indicates the incidence risk ratios in the age groups of a=1,…,20 and the regions i=1,…,96 in the years of t=1,…,14. $Y_{ait}$ represents the actual number of hepatitis B cases in the age group of a and region i age groups in the year of t, $E_{ait}$ represents the product of the expected number of cases in the age group of a and region i in the year of t, the number of cases in the region i and the incidence rate of the whole Xinjiang in the year of t. Compared with the number of cases, this index takes into account the population distribution characteristics. Compared with the commonly used index incidence rate, the risk of hepatitis B further reflects the actual risk of hepatitis B in 96 districts and counties, which can increase the comparability of 96 districts and counties.

**Figure 1:**
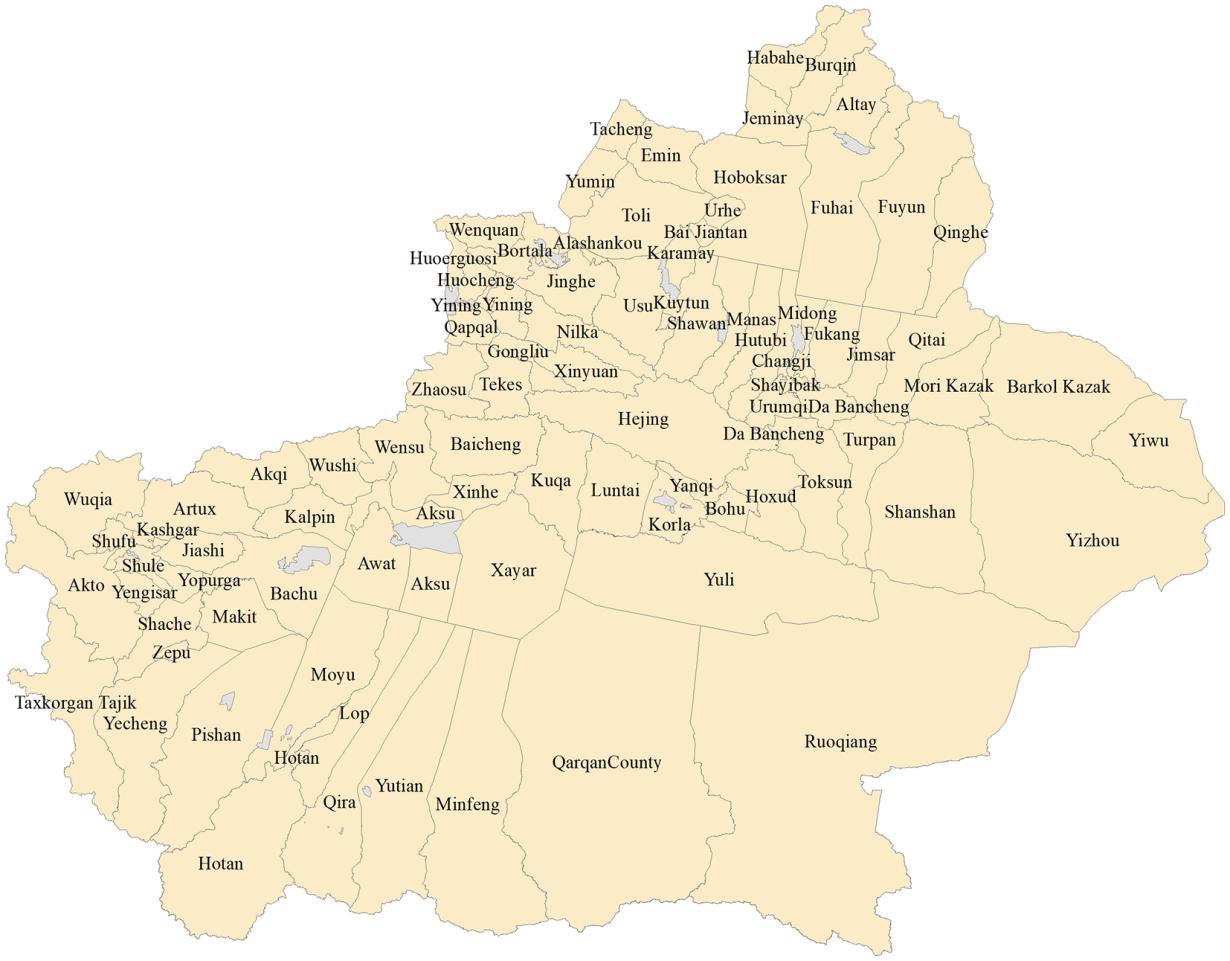
Map of 96 Districts and Counties in Xinjiang.

With the exception of Shihezi City, Alar City, Tumushuk City, Wujiaqu City, Beitun City, Tiemenguan City, Shuanghe City, Kokdala City, Kunyu City, and Huyanghe City, all 96 districts and counties in Xinjiang are the subject of this study’s analysis. Figure 1 depicts their locations. In every following map the next, the gray regions are designated as non-research areas whereas the yellow areas are research areas.

### 2.2. Statistical methods

#### 2.2.1. Global trend analysis

Global trend analysis is a three-dimensional perspective with a certain attribute value of data as height, which can reflect the main characteristics of changes in spatial areas. The primary components of a 3D perspective view are attribute values and spatial positions. A two-dimensional spatial rectangular coordinate system represents the geographic location together with the attribute values at the height of the location.

#### 2.2.2. Spatial autocorrelation analysis

The spatial situation of an attribute value across the entire region can be reflected by spatial autocorrelation (18). Within the study area, the method incorporates location and eigenvalues. Global and local spatial autocorrelation analyses are two subsets of spatial autocorrelation analysis. The most used statistic for analyzing global spatial autocorrelation, the global Moran’s I index, ranges from −1 to +1, indicating that the disease’s spatial distribution is either aggregated (>0), scattered (=0), or random (<0). In addition to accurately identifying geographical disparities brought on by spatial correlation, local spatial autocorrelation analysis may also pinpoint regions with a high prevalence of object properties. Four local spatial autocorrelation distributions are produced by the local Moran’s index: high-high, high-low, low-low, and low-high aggregation forms. The geographical distribution of the region and its neighbors as areas with a high attribute value is shown by the high-high aggregation form; The spatial distribution of locations with high attribute values and areas nearby with low attribute values is represented by the high-low clustering forms; The low-high aggregate form represents the spatial distribution of areas with low attribute values and regions adjacent to them that have high attribute values; The low-low aggregation form represents the spatial distribution of the area and its neighbors as areas with low attribute values.

#### 2.2.3. Statistical analysis of spatio-temporal scanning

The trend of spatial aggregation over time cannot be identified by spatial autocorrelation methods. Spatio-temporal scanning solves this problem. This method can not only reflect the trend of spatial clustering with time but also get the relative danger of the clustering region, locating the location of the spatial clustering region more accurately (19). The analysis process includes the following steps: firstly, any geographical position is randomly selected as the bottom center of the scanning window, and the corresponding geographical area and corresponding time interval are constantly changing until the specified upper limit position is reached. Secondly, for each scanning window, the expected incidence is calculated based on the actual incidence and population, and then the Log Likelihood Ratio (LLR) statistic is constructed by using the actual and expected values outside the scanning window, and the window with the largest LLR is selected as the high clustering window. Finally, the clustering test is conducted to obtain statistically significant results.

#### 2.2.4. Spatial age-period-cohort model

Assume that *Y*_*it*_, the number of hepatitis B cases in the age group a in the year *t*, follows a Poisson distribution with a mean $\mu_{at}=E_{at}SMR_{at}$, where *μ*_*at*_ indicates the mean value in the age group of a in the year of *t*, *E*_*at*_ represents the product of the expected number of cases in the age group of a in the year of *t*, *SMR*_*at*_ indicates the incidence risk ratios in the age group of a in the year of *t*. Taking the characteristics of patients with hepatitis B into consideration, a model with age, period and cohort effect was constructed, which was proposed by Leroux (20), and its expression is as follows.


$$Y_{at}∼Poisson\left(\mu_{at}=E_{at}SMR_{at}\right)$$

$$\log SMR_{at}=\beta +\gamma_{a}+\alpha_{t}+\vartheta_{k}$$

Where β is an overall rate, $\gamma_{a}$ is the age effect, $\alpha_{t}$ is the period effect, and $\vartheta_{k}$ is the cohort effect, and k=M×(A−a)+t, wherein, A=20 stands for the number of age groups, the age group interval is five times as wide as the period’s interval (21, 22), therefore, M=5. Since prior studies have shown that the incidence of hepatitis B has spatio-temporal characteristics, the only way to identify the high-incidence regions is to thoroughly investigate the spatio-temporal distribution features of hepatitis B. The introduction of space and spatio-temporal effects are considered in this study (13) to fully understand the epidemic characteristics of hepatitis B. This study considers the following models are considered herein to explore the optimal one.


$$\log SMR_{ait}=\beta +\gamma_{a}+\alpha_{t}+\vartheta_{k}+\phi_{i}+u_{i}$$

$$\log SMR_{ait}=\beta +\gamma_{a}+\alpha_{t}+\vartheta_{k}+\phi_{i}+\delta_{it}$$

$$\log SMR_{ait}=\beta +\gamma_{a}+\alpha_{t}+\vartheta_{k}+\phi_{i}+u_{i}+\delta_{it}$$

The above formulas are recorded as model 2, model 3, and model 4 respectively, where $\;\phi_{i}$ obeys a conditional autoregressive distribution (23), which denotes the existence of a spatial structure effect, indicating that the unidentified characteristic on the ith region has a geographic structure, and reflects the existence of spatial autocorrelation in the neighboring spatial of each district and county; $u_{i}$ follows a normal distribution, indicating a spatially unstructured effect; it represents an unknown feature in the ith region that lacks spatial structure and may also reflect other random effects brought on by non-spatial variables; The random effect of spatio-temporal interactions $\delta_{it}$, which obeys a normal distribution, demonstrated that the spatial organization has no impact on the trend of each district’s and county’s incidence over time (24). Using the R-INLA^1^ implementation of the model, the model was fitted and inferred using the Bayesian-INLA approach. The model was chosen using the DIC criterion (25), which allows a combination in the accuracy of fit and complexity of the model.

#### 2.2.5. Bayesian-INLA method

INLA method is a Bayesian fast calculation method proposed by Rue et al. (16). For hidden Gaussian model of Gaussian Markov random field. The hidden Gaussian model includes a large class of statistical models, namely the generalized additive model. Firstly, the generalized additive regression model is represented by a three-stage hierarchical modeling method, and the marginal distribution of parameters and hyperparameters in the model is obtained by Bayesian statistics. Finally, two marginal distributions are calculated by the INLA method, and the main steps are as follows: the first step is to expand the hyperparametric marginal posterior distribution; the quasi-Newton algorithm is used to obtain the modes of the hyperparameter posterior, the nodes of numerical integration are selected by the points calculated with the modes as the center of the mode-centered composite design (CCD) method, and the marginal posterior distribution of the hyperparameter is calculated by the numerical integration of the interpolation function. In the second step, Gaussian approximation, Laplace approximation, or simplified Laplace approximation method are used to approximate the marginal distribution of parameters. Compared with the Gaussian approximation method, the simplified Laplace approximation method is characterized by high efficiency and precision, and it is the default method in the R-INLA package. The third step combines the first two steps through numerical integration. Refer to Rue et al. (16) for a more detailed introduction to the INLA algorithm.

## 3. Results

### 3.1. Descriptive results

In Xinjiang, there were 545,943 new cases of hepatitis B from 2006 to 2009, with no obvious upward or downward trend in the number of hepatitis B infections during the study period. Among the 96 districts and counties, the number of new cases in Tianshan District, Shayibak District, Xinshi District, Shuimogou District, Korla City, Kashi City, Hotan City, and Yining City was greater than the average number of cases from 2006 to 2019, among which Xinshi District of Urumqi City had the largest number of new cases from 2006 to 2017, and Kashi City had the highest from 2018 to 2019. Taking the incidence risk as an indicator, the highest risk of hepatitis B was found in the Xinshi District of Urumqi from 2006 to 2015 and 2017, in Korla City in 2016 and 2018, and in Kashi City in 2019.

**Figure 2:**
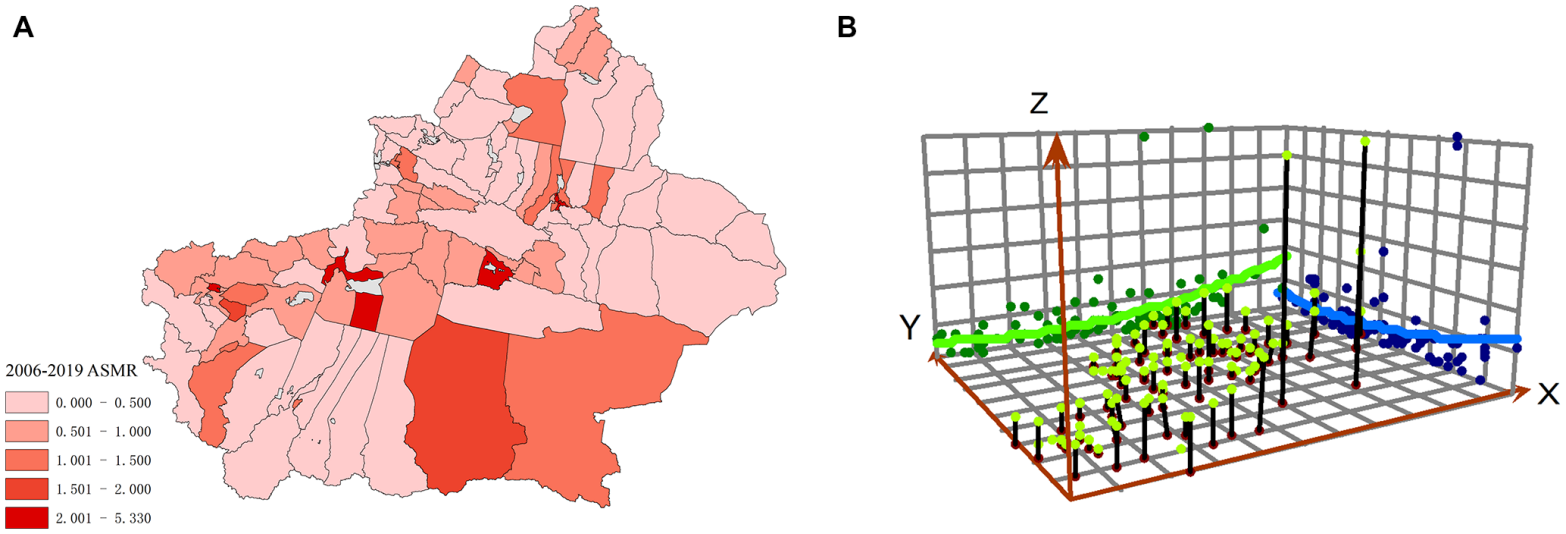
Average SMR **(A)** and spatial trend analysis chart **(B)** in Xinjiang.

Figure 2 shows the average SMR (ASMR) and spatial trend analysis for all districts in Xinjiang. The X-axis points east, the green curve represents the east–west direction, the Y-axis points north, the blue curve reflects the north–south direction, and the Z-axis represents the risk value, which is indicated by the height of the pole. The risk of hepatitis B is rising in Xinjiang from the west to the east and from the north to the south, displaying a clear ladder distribution.

### 3.2. Spatial clustering analysis results

#### 3.2.1. Global and local spatial autocorrelation

##### 3.2.1.1. Global spatial autocorrelation

The global autocorrelation analysis results of hepatitis B incidence risk in Xinjiang in 2006, 2008–2015, and 2017–2019 show that the global Moran’s I index of these years is greater than 0, and p<0.05,Z>1.96; therefore, there is a spatial positive correlation between the incidence risk of hepatitis B, with the global Moran’s I index ranging from 0.133 to 0.246 during the study period (Table 1). Among them, the global Moran’s I index is the smallest in 2017 (Moran’s I = 0.133), and the largest in 2019 (Moran’s I = 0.246).

**Table 1:** Global spatial autocorrelation analysis of Hepatitis B incidence in Xinjiang, 2006–2019.

Years	Moran’s I	*p*-Value	Z-Value
2006	0.187	0.005	3.215
2007	0.106	0.051	1.839
2008	0.154	0.015	2.588
2009	0.155	0.011	2.620
2010	0.190	0.007	3.120
2011	0.234	0.005	3.956
2012	0.157	0.024	2.661
2013	0.205	0.007	3.397
2014	0.228	0.005	3.665
2015	0.151	0.030	2.318
2016	0.099	0.053	1.726
2017	0.133	0.022	2.388
2018	0.188	0.005	3.143
2019	0.246	0.002	3.988

$\text{Mora}n^{\prime }s\;I>0$ and p<0.05 represent the clustered pattern of Hepatitis B.

##### 3.2.1.2. Local spatial autocorrelation

**Figure 3:**
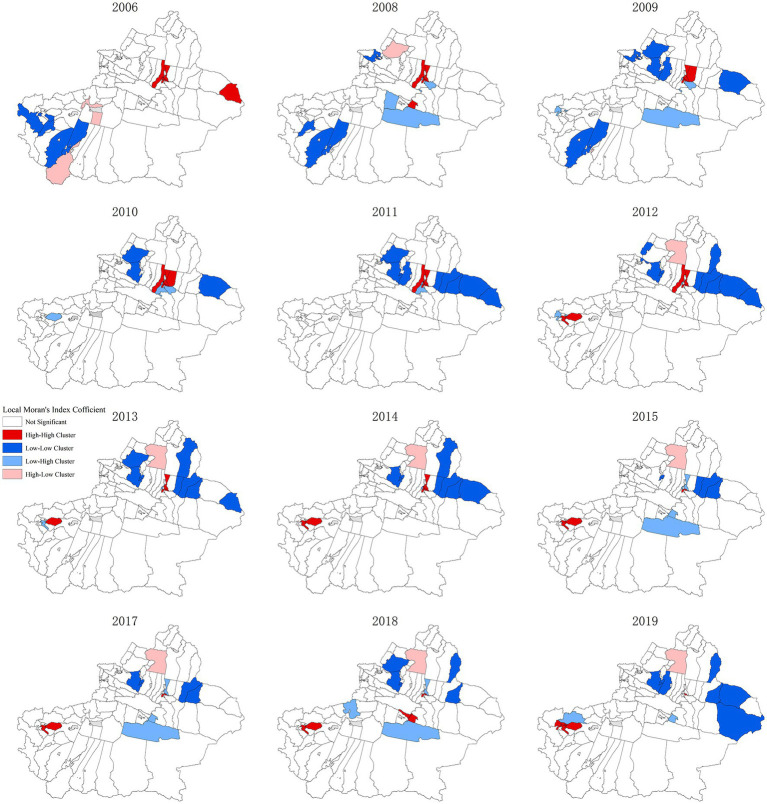
LISA cluster map of hepatitis B risk space in Xinjiang from 2006 to 2019.

The further local spatial autocorrelation analysis of 96 districts and counties can effectively detect the spatial differences caused by spatial correlation, and meanwhile, judge the spatial hot spots and high-incidence areas of the object attributes (the risk of hepatitis B herein). To judge whether the local correlation types and their clustering regions of 96 districts and counties are significant, the LISA clustering map will be exploited for further explanation (Figure 3). In the figure, the red regions indicate that the epidemic situation is in a high-high clustering mode, and the high-incidence area of hepatitis B epidemic, i.e., the epidemic hot spot area, and the blue regions indicates a low-low clustering mode, and the low-incidence area of hepatitis B epidemic, i.e., the epidemic cold spot area. It can be seen that the region shows more high-high and low-low forms of aggregation than the other two forms of aggregation. Shayibake District, Xinshi District, Shuimogou District, and Changji City are high-incidence areas of hepatitis B in most years. Midong District and Toutunhe District leapfrogged from high-high clustering regions to low-high epidemic areas in 2015, indicating that the risk of hepatitis B epidemic in Midong District and Toutunhe District decreased in 2015, while the nearby areas were still high epidemic areas. The same phenomenon exists in Bohu County, Bayinguoleng Mongolian Autonomous Prefecture. However, Jiashi County, Kashi Prefecture changed from a low-high clustering mode to a high-high clustering one. These epidemic hotspots are worthy of attention. In addition, Hoboksar Mongol Autonomous County, and Tacheng Prefecture were high-low clustering modes, and their surrounding counties are low-risk areas. Yuli County, Bayinguoleng Mongolian Autonomous Prefecture is a low-high clustering model, and its surrounding counties should be vigilant to strengthen epidemic control. Dushanzi District in Karamay, Barkol Kazak Autonomous County in Hami, Qitai County in Changji Hui Autonomous Prefecture, Jimsar County in Changji Hui Autonomous Prefecture, Mulei Kazak Autonomous County in Changji Hui Autonomous Prefecture, Yining City in Ili Kazakh Autonomous Prefecture and Wusu City in Tacheng were all cold spots. Yiwu County in Hami City changed from high-high clustering mode to low-low clustering mode, indicating that the area and its surrounding areas changed from high epidemic area to high epidemic area, indicating that the epidemic situation in this area has improved. The epidemic situation was also controlled in Toli County, Tacheng District, which changed from a high-low clustering mode to a low-low clustering mode in 2009.

#### 3.2.2. Statistical results of spatio-temporal scanning

**Figure 4:**
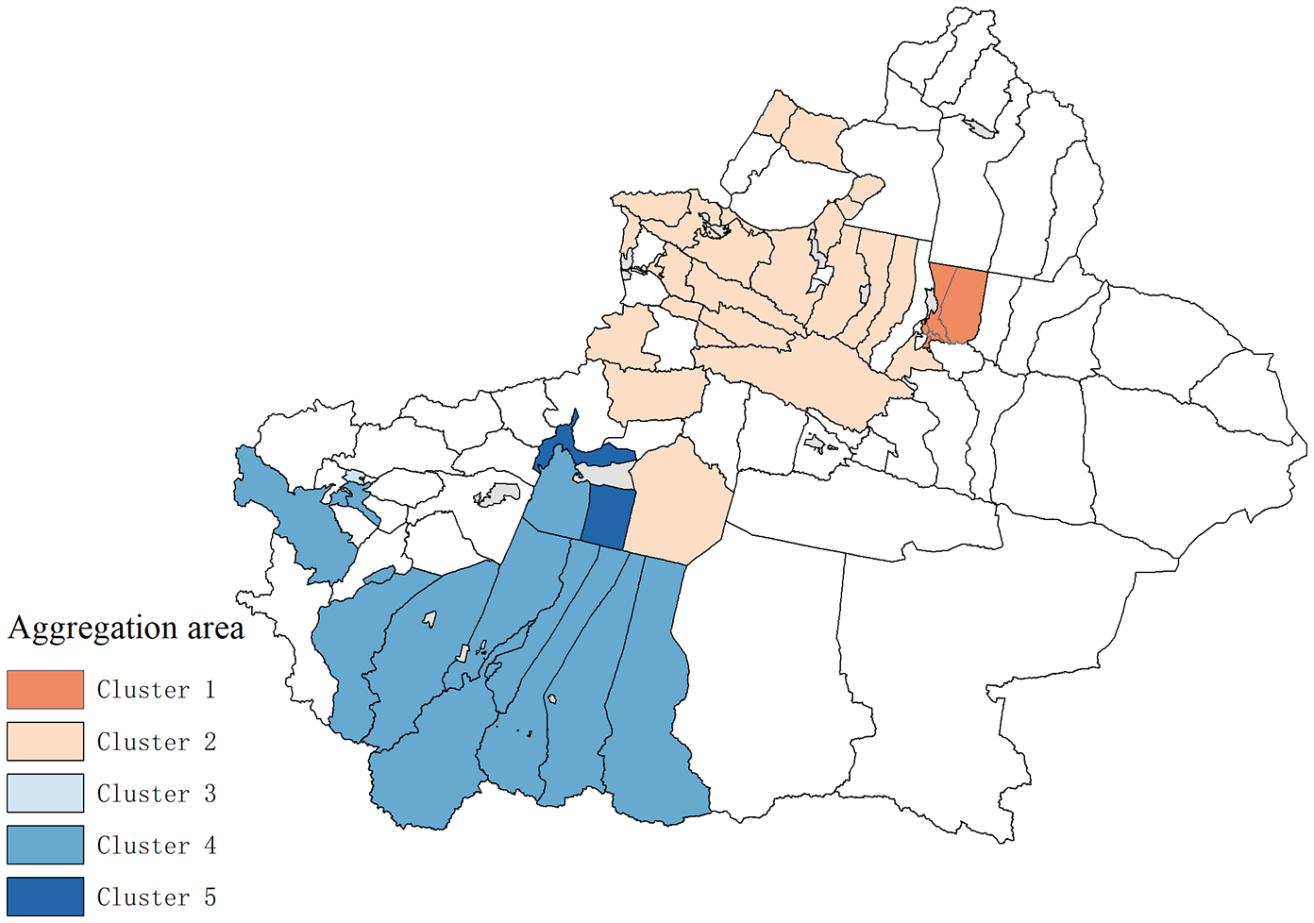
Spatio-temporal scanning clustering map of Hepatitis B incidence in Xinjiang from 2006.

The spatial–temporal scanning clustering results of hepatitis B risk in Xinjiang from 2006 to 2019 are shown in Figure 4 and Table 2. There are five types of clustering regions and the relative risk (RR) values of Level 1, 3, and 5 clustering regions are greater than 1, which can be classified as high clustering regions, while the RR values of Level 2, 4 clustering regions are less than 1, which can be classified as low clustering regions. Among Level 3 clustering regions, Kashi has the largest RR value in 2010–2016, reaching 4.51 times that of other areas. The log-likelihood ratio statistics (LLR) value can reflect the degree of clustering, and the larger the value, the stronger the clustering. That is, Level 1 clustering regions including Fukang City, Midong District, Shuimogou District, Xinshi District, and Tianshan District had the strongest clustering degree, and the RR value reached 4.49 in 2007–2013, 4.49 times that of other areas. p<0.001 of each epidemic clustering showed statistical significance.

**Table 2:** Results of spatio-temporal scan aggregation of Hepatitis B incidence in Xinjiang, from 2006 to 2019.

Cluster	IDs	Gathering time	Observed cases	Expected	RR	LLR	*p*
1	Fukang City, Midong District, Shui Mogou District, Xinshi District, Tianshan District	2007–2013	94,390	24,311	4.49	62895.91	<0.001
2	Bortala City, Alashankou City, Jinghe County, Nilka County, Yining County, Gongliu County, Xinyuan County, Usu City, Yining City, Wenquan County, Toli County, Kuytun City, Qapqal Xibe Autonomous County, Huocheng County, Dushanzi District, Tekes County, Yumin County, Karamay District, Huoerguosi City, Bai Jiantan District, Zhaosu County, Shawan County, Emin County, Tacheng City, Manas County, Urhe District, Kuqa County, Hutubi County, Baicheng County, Hoboksar Mongol Autonomous County, Luntai County, Xinhe County, Changji City, Tou Tunhe District, Xayar County, Hejing County, Urumqi County	2013–2019	38,315	95,232	0.36	25483.13	<0.001
3	Kashgar City	2010–2016	31,171	7,231	4.51	22144.49	<0.001
4	Hotan City, Hotan County, Moyu County, Pishan County, Lop County, Qira County, Yutian County, Yecheng County, Minfeng County, Zepu County, Shache County, Makit County, Yopurga County, Jiashi County, Bachu County, Yengisar County, Awat County, Kalpin County, Akto County, Shule County	2006–2011	28,620	60,771	0.44	11642.42	<0.001
5	Aksu City	2015–2019	14,673	4,902	3.05	6406.38	<0.001

RR, relative risk, LLR, Log Likelihood Ratio.

### 3.3. Spatial age-period-cohort results

Table 3 shows the comparison results of each model and the smaller the DIC index, the better. It can be seen from the results that the DIC of Model 4 is greatly reduced compared with Model 1, indicating that it is appropriate to introduce the spatial and spatio-temporal impact terms of hepatitis B incidence. Exploration and study based on Model 4 will be conducted next.

**Table 3:** Evaluation index of risk model of Hepatitis B incidence in 96 Districts of Xinjiang.

Model	DIC
$\log SMR_{at}=\beta +\gamma_{a}+\alpha_{t}+\vartheta_{k}$	846245.42
$\log SMR_{ait}=\beta +\gamma_{a}+\alpha_{t}+\vartheta_{k}+\phi_{i}+u_{i}$	354441.90
$\log SMR_{ait}=\beta +\gamma_{a}+\alpha_{t}+\vartheta_{k}+\phi_{i}+\delta_{it}$	325297.76
$\log SMR_{ait}=\beta +\gamma_{a}+\alpha_{t}+\vartheta_{k}+\phi_{i}+u_{i}+\delta_{it}$	325059.76

DIC: Deviation Information Criterion, with a smaller value the better.

**Figure 5:**
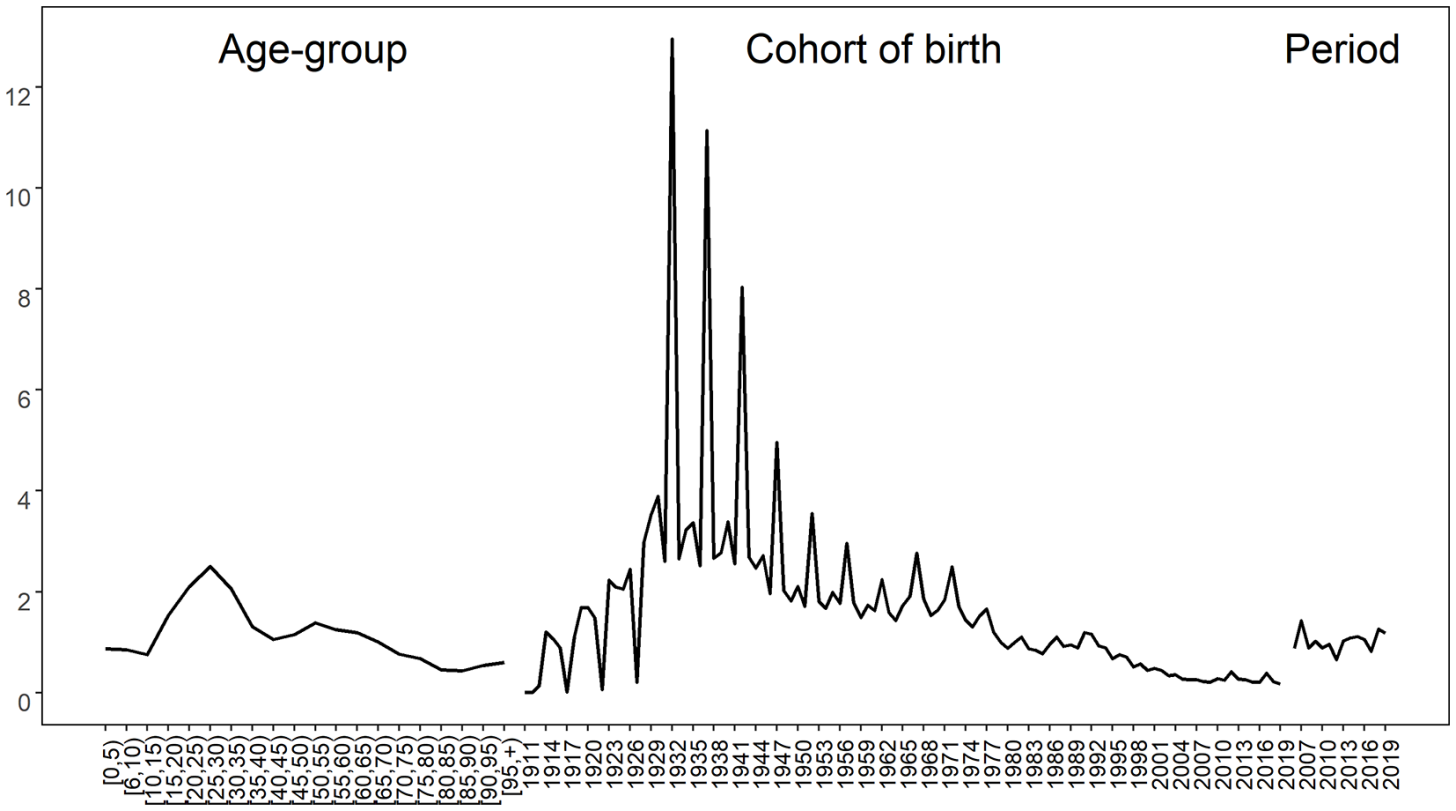
Average risk of Hepatitis B by age **(left)**, birth cohort **(middle)** and period **(right)** in Xinjiang.

**Figure 6:**
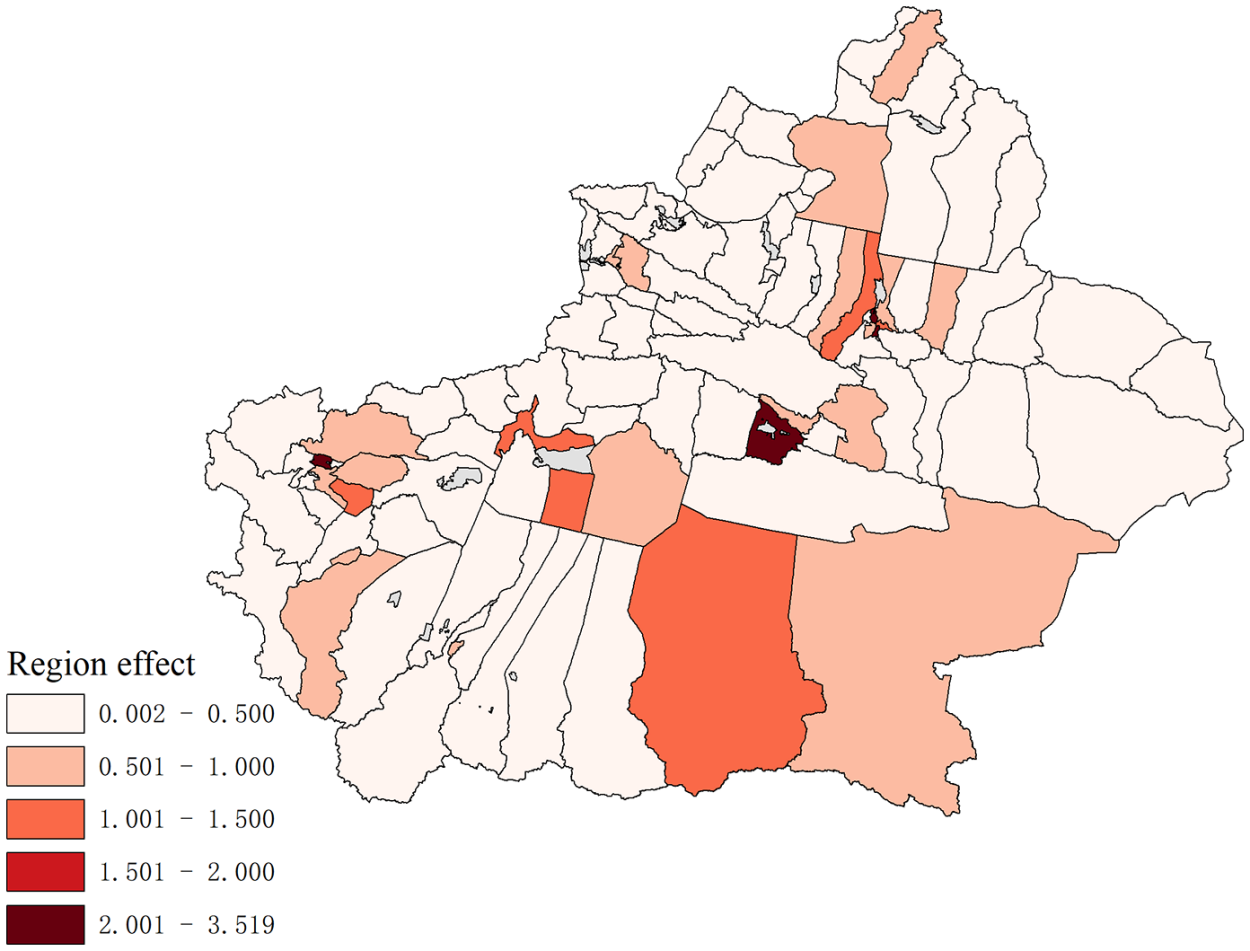
Hepatitis B risk of the total spatial effects in 96 Districts and Counties in Xinjiang.

**Figure 7:**
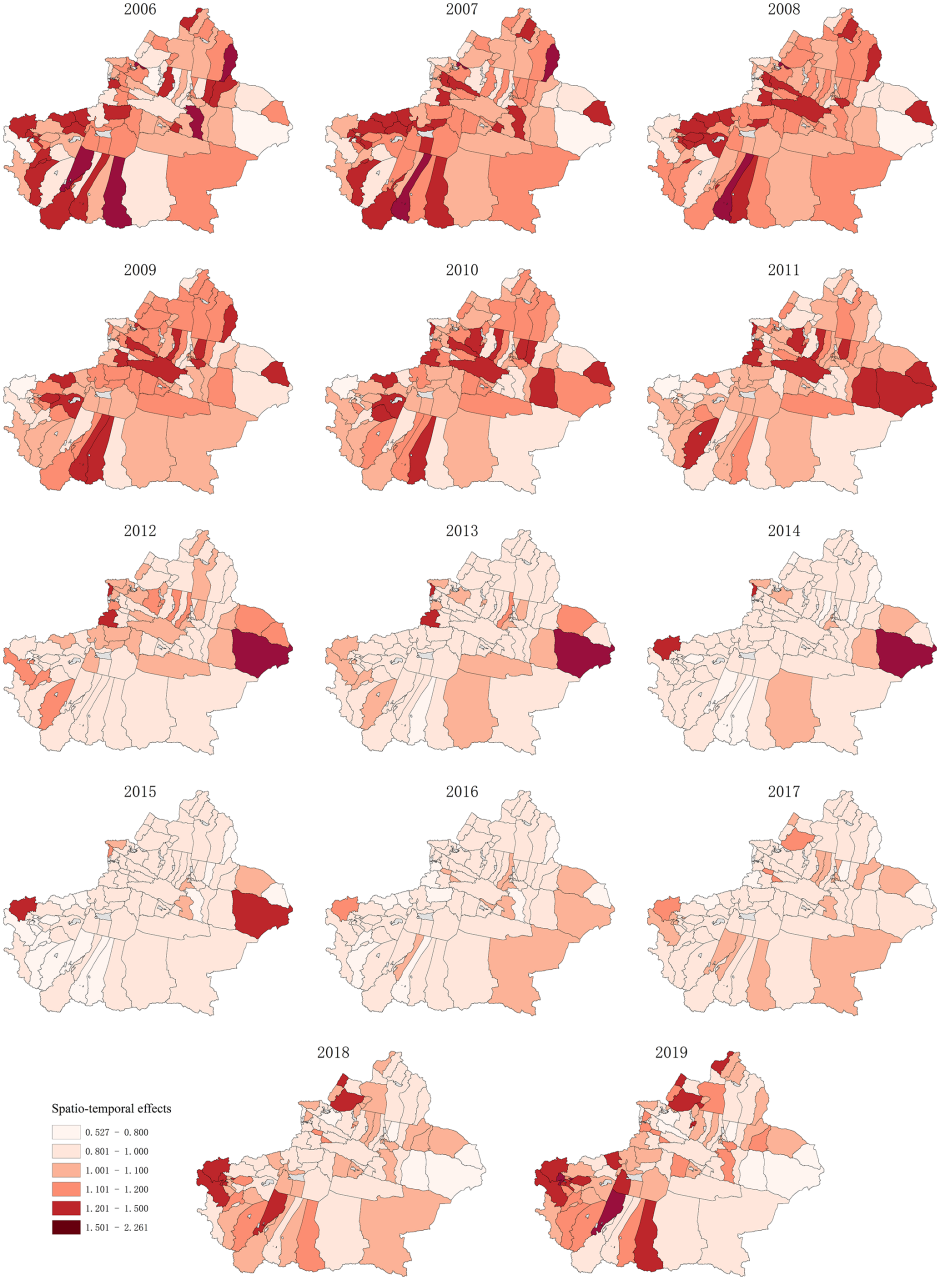
Hepatitis B risk of spatio-temporal effects in 96 Districts and Counties of Xinjiang from 2006 to 2019.

The results of the spatial age-period-cohort effect analysis of hepatitis B in Xinjiang from 2006 to 2019 are shown in Figures 5–7. The results show that the average incidence risk of hepatitis B does not show a single increase or decrease trend with the increase of age. From 0 to 15 years of age, it showed a decreasing trend; From 16 years of age on, it increased until reaching a peak at [25,30), after which it decreased; From 40 years of age on, it increased once more until reaching a second peak at [50,55), after which it again showed a decreasing trend. The average risk of hepatitis B fluctuated around 1 over time, reaching a maximum of 1.419 in 2007 and a minimum of 0.653 in 2012. The average incidence risk by birth cohort also had certain dynamics, displaying a trend of increasing first, then decreasing, and finally tending to be stable.

Figure 6 shows the hepatitis B risk of the total spatial effects of 96 districts and counties in Xinjiang from 2006 to 2019. The regions with high hepatitis B risk include Tianshan District, Xinshi District, Shuimogou District, and Changji City in northern Xinjiang, Aksu, Kashi City, Korla City, Qiemo County, and Yopurga County in southern Xinjiang. Among them, the risks of hepatitis B in Tianshan District, Xinshi District, Korla City, and Kashi City were greater than 2, of which the incidence risk in Xinshi District was the highest, up to 3.519.

The spatio-temporal effect is used to express changes that cannot be reflected separately except the spacing effect and time effect. The spatio-temporal interaction effect established in this study shows that the influence of unobserved variables on the risk of hepatitis B can be separated into time and space effects. The function of this variable is to find out whether there are any unobserved variables affecting 96 districts and counties in Xinjiang. Figure 7 shows the hepatitis B risk of spatio-temporal effect in 96 districts and counties of Xinjiang from 2006 to 2019. The spatio-temporal effect on the hepatitis B risk in adjacent areas through time can be seen in the figure. Taking Toli County and Habahe County as examples, the changes in spatio-temporal effects in the two regions from 2006 to 2019 were observed. The spatio-temporal effects of Tori County and Habahe County showed an increasing trend from 2006 to 2019, indicating that unobserved variables had a greater impact on Tori County and Habahe County, such as local medical and health conditions and social and economic conditions.

## 4. Discussion

The trend analysis, spatial autocorrelation analysis, and spatio-temporal scanning statistical methods were used in this study to explore the hepatitis B risk in 96 districts and counties of Xinjiang from 2006 to 2019, and a spatial age-period-cohort model was established considering the effects of space, age, period and birth cohort to analyze the incidence trend of hepatitis B in Xinjiang in recent years more comprehensively and deeply.

The spatial autocorrelation results from 2006 to 2019 show a positive spatial correlation in the distribution of hepatitis B in the years except 2007 and 2016, with the year 2019 having the strongest spatial correlation degree. Shayibak District, Xinshi District, Shuimogou District, and the Changji City of Urumqi were high-incidence regions of hepatitis B in most years, and Qitai County, Jimsar County, Mulei Kazak Autonomous County, Yining City, Wusu City were epidemic cold spots. This may be related to population density, medical and health conditions, and social economy.

Spatial–temporal clustering scanning analysis shows five types of clustering regions, and the relative risk degree values of Level 1, 3, and 5 clustering regions were greater than 1. Among them, the RR value of the Level 3 clustering region, Kashi City was the largest in 2010–2016, which is 4.51 times that of other regions. Fukang City, Midong District, Shuimogou District, Xinshi District, and Tianshan District are Level 1 clustering regions, and their RR value was only smaller than that of Kashi. The clustering time was from 2007 to 2013, and their RR value was 4.49. The relative risk of Level 2 and 4 clustering regions was low.

A spatial age-period-cohort study found differences in the risk of hepatitis B among different age groups. The average incidence risk is higher at the ages of 25–30 and 50–55. This may be related to the transmission route of hepatitis B, which mainly include iatrogenic transmission, mother-fetus transmission, father-infant transmission, sexual contact transmission, and daily contact transmission (26). The main transmission modes among people aged 25–30 are iatrogenic contact transmission and daily contact transmission. As the content of HBV is the highest in blood, the transmission of HBV through blood is one of the main ways, and the virus is likely to spread in the process of blood transfusion, injection, surgery, tattoo, ear piercing, and tooth extraction. In sexual transmission, especially those with multiple sexual partners, the risk of HBV infection will increase. Daily close contact and communication mainly occur in families and public places. These may be the reasons for the high risk of hepatitis B in this age group. For the population aged 50–55, this may be related to the changes in physical function brought about by age. This suggests that we need to carry out more basic research among high-risk groups for better preventive measures. In addition, it was found in this study that the risk of hepatitis B at the age of 0–15 was low and showed a downward trend, which may be related to the vaccination of the hepatitis B vaccine. Perinatal mother–child transmission is the most common route of HBV transmission, that is, HBV is transmitted to infants through the blood and body fluids of HBV-positive mothers, while postpartum breastfeeding and close contact can transmit HBV horizontally. Mother–child transmission is the main route of children’s infection (27); therefore, it is particularly important to prevent HBV transmission caused by mother–child transmission. Hepatitis B vaccination is an important tool and effective measure for the prevention and control of the hepatitis B epidemic (28). To control hepatitis B virus infection, the World Health Organization formulated a global plan to control hepatitis B in 2010 (29). The strategic plan is divided into three stages. In the first stage from 1989 to 1993, countries must investigate and confirm the prevalence and control of hepatitis B in their populations, and choose to establish vaccination procedures for the hepatitis B vaccine. The second stage is from 1994 to 2000, and all newborns and children must be vaccinated in a planned way. The key point of this stage is to improve the immunity of the hepatitis B vaccine, reduce the vaccination times as much as possible, and determine whether it is necessary to increase the vaccination measurement for a special population. The third stage is 2000–2010, in which newborns, children, and high-risk groups are the key targets of vaccination, and will continue to be vaccinated. The implementation of this plan has promoted the vaccination of the hepatitis B vaccine, and as a result, the risk of hepatitis B in newborns and children is low.

The period effects usually show that historical events and factors have a direct impact on the incidence (30, 31), and the fluctuation of the hepatitis B risk may be caused by improved medical intervention. The average risk of hepatitis B fluctuated around 1 over time, reaching a peak of 1.419 in 2007 and a minimum in 2012. Cohort effects reflect the changes in lifestyle and risk factors exposure at different times (32, 33). The increase in the birth cohort presents certain dynamics, which is manifested in the trend of increasing first, then decreasing and finally stabilizing. This may be related to the popularization of the hepatitis B vaccine in the hepatitis B prevention strategy, and this change in the birth cohort may also benefit from the improvement of living conditions and medical levels.

Combined with age, period, and cohort effect, it is found that the areas with a high risk of hepatitis B are Tianshan District, Xinshi District, Shuimogou District, Changji City in northern Xinjiang, Aksu City, Kashi City, Korla City, Qiemo County and Yuepuhu County in southern Xinjiang. Urumqi is a prefecture-level city and the capital of Xinjiang Uygur Autonomous Region, and it is also an important comprehensive transportation hub city. Urumqi has seven municipal districts, three of which are high-incidence regions of the hepatitis B epidemic, and Changji City, Aksu City, Kashi City, and Korla City are the central cities of their local prefectures. Affected by urbanization, the economic development in these regions is relatively fast, and the people’s living standards are constantly improving, which leads to an increase in population mobility and easier access to hepatitis B patients; therefore the incidence risk in these regions is high. It is found by the spatio-temporal effect term that there are unobserved variables affecting the incidence of hepatitis B in some districts and counties of Xinjiang.

This study may have some limitations. We have assessed the risk of hepatitis B, but previous research shows that the incidence of hepatitis B displays gender difference, and the influence of gender factors on the results is not considered when analyzing the risk factors related to hepatitis B. In addition, HBV infection in high-risk groups needs to be known to better prevent HBV infection.

## 5. Conclusion

There was a positive spatial correlation and a spatial–temporal correlation in the risk of hepatitis B incidence in Xinjiang, it tends to grow from west to east and from north to south. Hepatitis B risk was influenced by age, period, cohort, and also by unobserved factors. Among the 96 districts and counties, the risk of incidence was highest in Tianshan District, Xinshi District, Shuimogou District, Changji City, Aksu City, Kashi City, Korla City, Qiemo County, and Yuepuhu County.

## Data availability statement

The original contributions presented in the study are included in the article/supplementary material, further inquiries can be directed to the corresponding authors.

## Author contributions

YW: writing—original draft and data analysis. NX, FL, and ZW: data analysis, reviewing, and editing. SD and KW: data analysis and revised manuscript. XH: conception, reviewing, and editing. All authors contributed to the article and approved the submitted version.

## Funding

This research was funded by the National Natural Science Foundation of China (Grant Nos. 11961065 and 11961071), Youth Science and Technology Innovation Talent of Tianshan Talent Training Program in Xinjiang (Grant No. 2022TSYCCX0099), and the Chinese Foundation for Hepatitis Prevention and Control (YGFK20200059).

## Conflict of interest

The authors declare that the research was conducted in the absence of any commercial or financial relationships that could be construed as a potential conflict of interest.

## Publisher’s note

All claims expressed in this article are solely those of the authors and do not necessarily represent those of their affiliated organizations, or those of the publisher, the editors and the reviewers. Any product that may be evaluated in this article, or claim that may be made by its manufacturer, is not guaranteed or endorsed by the publisher.

## Footnotes

^1^ www.r-inla.org
